# *N*,*N*′-Bis(4-methyl­benzyl­idene)benzene-1,4-diamine

**DOI:** 10.1107/S1600536811041675

**Published:** 2011-10-12

**Authors:** Haoyang Li, Yonggang Xiang, Hongfei Han

**Affiliations:** a Department of Chemistry, Taiyuan Normal University, Taiyuan 030031, People’s Republic of China

## Abstract

The centrosymmetric title compound, C_22_H_20_N_2_, crystallizes with one half-mol­ecule in the asymmetric unit. The dihedral angle between the central and outer benzene rings is 46.2 (2)°.

## Related literature

For the use of Schiff bases as ligands in metal complexes, see: Chen *et al.* (2008 ▶); May *et al.* (2004 ▶).
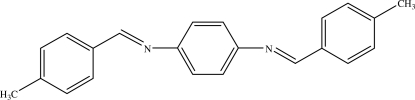


## Experimental

### 

#### Crystal data

C_22_H_20_N_2_
*M* *_r_* = 312.40
Monoclinic, 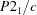

*a* = 6.4750 (6) Å
*b* = 7.1561 (8) Å
*c* = 19.594 (2) Å
β = 107.555 (1)°
*V* = 865.61 (16) Å^3^
*Z* = 2
Mo *K*α radiation
μ = 0.07 mm^−1^
*T* = 293 K
0.46 × 0.40 × 0.37 mm

#### Data collection

Bruker SMART CCD area-detector diffractometer
Absorption correction: multi-scan (*SADABS*; Sheldrick, 1996 ▶) *T*_min_ = 0.968, *T*_max_ = 0.974
4151 measured reflections
1532 independent reflections
844 reflections with *I* > 2σ(*I*)
*R*_int_ = 0.034

#### Refinement

*R*[*F* ^2^ > 2σ(*F* ^2^)] = 0.044
*wR*(*F* ^2^) = 0.145
*S* = 1.04
1532 reflections
111 parameters
H-atom parameters constrained
Δρ_max_ = 0.13 e Å^−3^
Δρ_min_ = −0.17 e Å^−3^

### 

Data collection: *SMART* (Bruker, 2007 ▶); cell refinement: *SAINT* (Bruker, 2007 ▶); data reduction: *SAINT*; program(s) used to solve structure: *SHELXS97* (Sheldrick, 2008 ▶); program(s) used to refine structure: *SHELXL97* (Sheldrick, 2008 ▶); molecular graphics: *SHELXTL* (Sheldrick, 2008 ▶); software used to prepare material for publication: *SHELXTL*.

## Supplementary Material

Crystal structure: contains datablock(s) I, global. DOI: 10.1107/S1600536811041675/bt5670sup1.cif

Structure factors: contains datablock(s) I. DOI: 10.1107/S1600536811041675/bt5670Isup2.hkl

Supplementary material file. DOI: 10.1107/S1600536811041675/bt5670Isup3.cml

Additional supplementary materials:  crystallographic information; 3D view; checkCIF report

## Figures and Tables

**Fig. 1.**
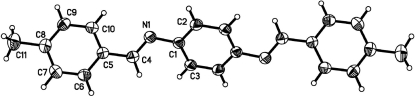
The molecular structure, showing the atom–numbering scheme. Displacement ellipsoids are drawn at the 30% probability level. H atoms have been omitted for clarity.

## supplementary crystallographic information

### Comment

Schiff bases containing the C═N bond have been receiving considerable attention for many years, primarily due to their importance as ligands in metal complexes with special biological (May *et al.*, 2004),   and catalytic properties (Chen *et al.*, 2008).

As a part of our studies on synthesis and structural peculiarities of Schiff bases derived from 1,4-benzenediamine and 4-methyl benzaldehyde, we determined the structure of the title compound (Fig. 1). The molecule includes two C═ N bonds, which are coplanar. The distance between the C atom and the N atom in the C═N bond is 1.266 (2) Å. In the structure the dihedral angle between adjacent benzene rings planes is 46.2 (2)°.

### Experimental

1,4-benzenediamine (0.324 g, 3 mmol) was added dropwise with stirring at 273K to a solution of 4-methyl benzaldehyde (0.721 g, 6 mmol) in ethanol. The mixture were warmed to room temperature and stirred for 2 h. The reaction mixture was filtered and the filter cake was recrystallized from ethanol (yield 75%). Crystals of the title compound suitable for X-ray diffraction were obtained by slow evaporation of a tetrahydrofuran solution.

### Refinement

All H atoms were positioned geometrically (C—H = 0.93–0.96 Å), and refined as riding with *U*_iso_(H) = 1.2*U*_eq_ or 1.5*U*_eq_ (methyl H atoms).

### Crystal data

**Table:**

C_22_H_20_N_2_	*F*(000) = 332
*M**_r_* = 312.40	*D*_x_ = 1.199 Mg m^−^^3^
Monoclinic, *P*2_1_/*c*	Mo *K*α radiation, λ = 0.71073 Å
*a* = 6.4750 (6) Å	Cell parameters from 954 reflections
*b* = 7.1561 (8) Å	θ = 2.9–23.3°
*c* = 19.594 (2) Å	µ = 0.07 mm^−^^1^
β = 107.555 (1)°	*T* = 293 K
*V* = 865.61 (16) Å^3^	Block, colorless
*Z* = 2	0.46 × 0.40 × 0.37 mm

### Data collection

**Table:**

Bruker SMART CCD area-detector diffractometer	1532 independent reflections
Radiation source: fine-focus sealed tube	844 reflections with *I* > 2σ(*I*)
graphite	*R*_int_ = 0.034
φ and ω scans	θ_max_ = 25.0°, θ_min_ = 3.1°
Absorption correction: multi-scan (*SADABS*; Sheldrick, 1996)	*h* = −7→7
*T*_min_ = 0.968, *T*_max_ = 0.974	*k* = −8→6
4151 measured reflections	*l* = −23→23

### Refinement

**Table:**

Refinement on *F*^2^	Secondary atom site location: difference Fourier map
Least-squares matrix: full	Hydrogen site location: inferred from neighbouring sites
*R*[*F*^2^ > 2σ(*F*^2^)] = 0.044	H-atom parameters constrained
*wR*(*F*^2^) = 0.145	*w* = 1/[σ^2^(*F*_o_^2^) + (0.0581*P*)^2^ + 0.1422*P*] where *P* = (*F*_o_^2^ + 2*F*_c_^2^)/3
*S* = 1.04	(Δ/σ)_max_ < 0.001
1532 reflections	Δρ_max_ = 0.13 e Å^−^^3^
111 parameters	Δρ_min_ = −0.17 e Å^−^^3^
0 restraints	Extinction correction: *SHELXL97* (Sheldrick, 2008), Fc^*^=kFc[1+0.001xFc^2^λ^3^/sin(2θ)]^-1/4^
Primary atom site location: structure-invariant direct methods	Extinction coefficient: 0.069 (8)

### Special details

**Table:**

Geometry. All e.s.d.'s (except the e.s.d. in the dihedral angle between two l.s. planes) are estimated using the full covariance matrix. The cell e.s.d.'s are taken into account individually in the estimation of e.s.d.'s in distances, angles and torsion angles; correlations between e.s.d.'s in cell parameters are only used when they are defined by crystal symmetry. An approximate (isotropic) treatment of cell e.s.d.'s is used for estimating e.s.d.'s involving l.s. planes.
Refinement. Refinement of *F*^2^ against ALL reflections. The weighted *R*-factor *wR* and goodness of fit *S* are based on *F*^2^, conventional *R*-factors *R* are based on *F*, with *F* set to zero for negative *F*^2^. The threshold expression of *F*^2^ > σ(*F*^2^) is used only for calculating *R*-factors(gt) *etc*. and is not relevant to the choice of reflections for refinement. *R*-factors based on *F*^2^ are statistically about twice as large as those based on *F*, and *R*- factors based on ALL data will be even larger.

### Fractional atomic coordinates and isotropic or equivalent isotropic displacement parameters (Å^2^)

**Table:**

	*x*	*y*	*z*	*U*_iso_*/*U*_eq_	
N1	0.3167 (3)	0.4579 (2)	0.61261 (9)	0.0604 (6)	
C1	0.4131 (3)	0.4803 (3)	0.55707 (11)	0.0543 (6)	
C2	0.2944 (3)	0.5650 (3)	0.49390 (11)	0.0604 (6)	
H2	0.1554	0.6084	0.4891	0.072*	
C3	0.6190 (3)	0.4146 (3)	0.56201 (11)	0.0602 (6)	
H3	0.7002	0.3558	0.6038	0.072*	
C4	0.4309 (4)	0.4832 (3)	0.67687 (11)	0.0558 (6)	
H4	0.5734	0.5226	0.6857	0.067*	
C5	0.3491 (3)	0.4533 (3)	0.73774 (11)	0.0504 (5)	
C6	0.4766 (4)	0.4939 (3)	0.80622 (11)	0.0609 (6)	
H6	0.6167	0.5381	0.8135	0.073*	
C7	0.3996 (4)	0.4699 (3)	0.86443 (12)	0.0678 (7)	
H7	0.4889	0.4982	0.9101	0.081*	
C8	0.1925 (4)	0.4049 (3)	0.85571 (12)	0.0621 (6)	
C9	0.0668 (4)	0.3596 (3)	0.78732 (13)	0.0645 (6)	
H9	−0.0723	0.3134	0.7802	0.077*	
C10	0.1436 (3)	0.3814 (3)	0.72947 (12)	0.0609 (6)	
H10	0.0564	0.3476	0.6841	0.073*	
C11	0.1042 (5)	0.3887 (4)	0.91853 (13)	0.0913 (9)	
H11A	0.2211	0.3657	0.9614	0.137*	
H11B	0.0028	0.2872	0.9105	0.137*	
H11C	0.0326	0.5030	0.9236	0.137*	

### Atomic displacement parameters (Å^2^)

**Table:**

	*U*^11^	*U*^22^	*U*^33^	*U*^12^	*U*^13^	*U*^23^
N1	0.0561 (12)	0.0671 (13)	0.0566 (11)	0.0008 (9)	0.0150 (10)	0.0079 (9)
C1	0.0510 (14)	0.0562 (13)	0.0538 (13)	−0.0039 (10)	0.0131 (11)	0.0040 (10)
C2	0.0483 (13)	0.0689 (15)	0.0620 (14)	0.0065 (10)	0.0137 (11)	0.0091 (11)
C3	0.0513 (14)	0.0707 (15)	0.0540 (13)	0.0037 (11)	0.0090 (11)	0.0118 (11)
C4	0.0535 (14)	0.0499 (13)	0.0631 (14)	−0.0004 (10)	0.0162 (12)	0.0037 (10)
C5	0.0527 (13)	0.0424 (11)	0.0553 (13)	0.0031 (9)	0.0153 (11)	0.0031 (9)
C6	0.0574 (14)	0.0571 (14)	0.0659 (15)	−0.0033 (10)	0.0151 (12)	−0.0020 (11)
C7	0.0766 (17)	0.0665 (15)	0.0560 (14)	0.0031 (12)	0.0137 (13)	−0.0026 (11)
C8	0.0741 (16)	0.0499 (13)	0.0678 (15)	0.0103 (12)	0.0299 (13)	0.0089 (11)
C9	0.0597 (15)	0.0599 (14)	0.0774 (16)	−0.0007 (11)	0.0261 (13)	0.0070 (12)
C10	0.0555 (14)	0.0619 (14)	0.0609 (14)	−0.0018 (11)	0.0113 (11)	0.0006 (11)
C11	0.114 (2)	0.0928 (19)	0.0806 (18)	0.0107 (17)	0.0488 (17)	0.0172 (14)

### Geometric parameters (Å, °)

**Table:**

N1—C4	1.266 (2)	C6—C7	1.387 (3)
N1—C1	1.418 (3)	C6—H6	0.9300
C1—C2	1.384 (3)	C7—C8	1.380 (3)
C1—C3	1.389 (3)	C7—H7	0.9300
C2—C3^i^	1.381 (3)	C8—C9	1.380 (3)
C2—H2	0.9300	C8—C11	1.510 (3)
C3—C2^i^	1.381 (3)	C9—C10	1.377 (3)
C3—H3	0.9300	C9—H9	0.9300
C4—C5	1.459 (3)	C10—H10	0.9300
C4—H4	0.9300	C11—H11A	0.9600
C5—C6	1.378 (3)	C11—H11B	0.9600
C5—C10	1.390 (3)	C11—H11C	0.9600
			
C4—N1—C1	119.14 (19)	C8—C7—C6	121.1 (2)
C2—C1—C3	118.22 (19)	C8—C7—H7	119.4
C2—C1—N1	118.79 (19)	C6—C7—H7	119.4
C3—C1—N1	122.94 (19)	C7—C8—C9	117.8 (2)
C3^i^—C2—C1	120.5 (2)	C7—C8—C11	121.2 (2)
C3^i^—C2—H2	119.8	C9—C8—C11	121.1 (2)
C1—C2—H2	119.8	C10—C9—C8	121.3 (2)
C2^i^—C3—C1	121.3 (2)	C10—C9—H9	119.4
C2^i^—C3—H3	119.3	C8—C9—H9	119.4
C1—C3—H3	119.3	C9—C10—C5	121.1 (2)
N1—C4—C5	123.0 (2)	C9—C10—H10	119.4
N1—C4—H4	118.5	C5—C10—H10	119.4
C5—C4—H4	118.5	C8—C11—H11A	109.5
C6—C5—C10	117.6 (2)	C8—C11—H11B	109.5
C6—C5—C4	120.4 (2)	H11A—C11—H11B	109.5
C10—C5—C4	122.0 (2)	C8—C11—H11C	109.5
C5—C6—C7	121.1 (2)	H11A—C11—H11C	109.5
C5—C6—H6	119.5	H11B—C11—H11C	109.5
C7—C6—H6	119.5		
			
C4—N1—C1—C2	−140.7 (2)	C4—C5—C6—C7	178.63 (19)
C4—N1—C1—C3	41.9 (3)	C5—C6—C7—C8	−0.1 (3)
C3—C1—C2—C3^i^	−0.9 (3)	C6—C7—C8—C9	1.8 (3)
N1—C1—C2—C3^i^	−178.49 (19)	C6—C7—C8—C11	−176.6 (2)
C2—C1—C3—C2^i^	0.9 (4)	C7—C8—C9—C10	−1.1 (3)
N1—C1—C3—C2^i^	178.39 (19)	C11—C8—C9—C10	177.3 (2)
C1—N1—C4—C5	−176.51 (17)	C8—C9—C10—C5	−1.2 (3)
N1—C4—C5—C6	−176.06 (19)	C6—C5—C10—C9	2.8 (3)
N1—C4—C5—C10	4.8 (3)	C4—C5—C10—C9	−178.00 (19)
C10—C5—C6—C7	−2.2 (3)		

Symmetry codes: (i) −*x*+1, −*y*+1, −*z*+1.
